# Three-dimensional imaging for hepatobiliary and pancreatic diseases: Emphasis on clinical utility

**DOI:** 10.4103/0971-3026.45336

**Published:** 2009-02

**Authors:** Soo Jin Kim, Byung Ihn Choi, Se Hyung Kim, Jae Young Lee

**Affiliations:** 1 Department of Radiology and the Institute of Radiation Medicine, Seoul National University Hospital, 101 Daehangno, Jongno-gu, Seoul, 110-744, Korea; 2 Department of Radiology and the Institute of Radiation Medicine, Seoul National University College of Medicine, 101 Daehangno, Jongno-gu, Seoul, 110-744, Korea

**Keywords:** three-dimensional image, ultrasonography, CT, MRI

## Abstract

Three-dimensional (3D) imaging allows disease processes and anatomy to be better understood, both by radiologists as well as physicians and surgeons. 3D imaging can be performed with USG, CT scan and MRI, using different modes or rendering that include surface-shaded display, volume-based rendering, multiplanar imaging, etc. All these techniques are used variably depending on the indications.

Three-dimensional (3D) imaging has become a popular modality because of its ability to provide 3D views of the patient's anatomy. It also does not have many of the inherent shortcomings of two-dimensional (2D) imaging. Two-dimensional images make interpretation more difficult by forcing the reader to restructure the 2D images mentally in order to appreciate the true form of the disease or organ. Three-dimensional images, on the other hand, provide easier-to-understand information, while being more efficient, accurate, objective, and reproducible. Three-dimensional imaging is more photorealistic due to advanced rendering; allows any arbitrary plane to be obtained, which was not previously possible with 2D USG techniques; and increases patient throughout by offline rendering.

In this review, the clinical utility of 3D imaging with USG, CT scan, and MRI in hepatobiliary diseases will be discussed.

## Three-dimensional USG

### Three-dimensional rendering

The selection of the rendering technique used very often determines which information is transmitted to the operator by the 3D USG image display.[1] There are many techniques for displaying 3D images; they are divided into three classes: surface rendering, multiplanar viewing, and volume rendering.[2] The choice of the best rendering technique is generally determined by the clinical application.

**Figure 1:**
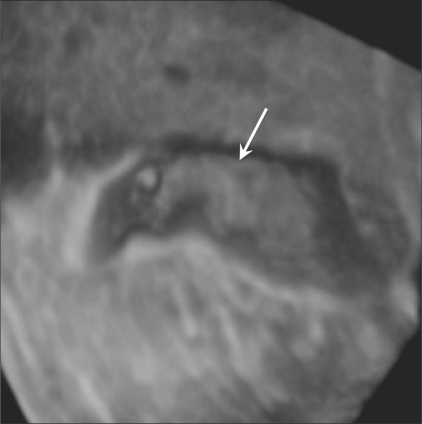
3-D surface-rendering image of a gallbladder polyp (arrow) in a 30-year-old man. On pathologic examination after cholecystectomy, this lesion was confirmed to be an adenomatous polyp

1. ***Surface-based rendering:*** The most commonly used 3D display technique is based on the visualization of surfaces of structures or organs. This technique can be performed manually, with the operator determining the boundaries of the structures, or by automated techniques.[3] After the tissues or structures have been classified, a surface-rendering algorithm shades and illuminates the surface representation, at times adding depth cues, so that topography and 3D geometry can be more easily comprehended. An example of 3D surface rendering is shown in Figure 1, which demonstrates a small polyp in a distended gallbladder. The operators may view the anatomy from different perspectives using either automatic rotation or user-controlled motion.

**Figure 2 (A-D):**
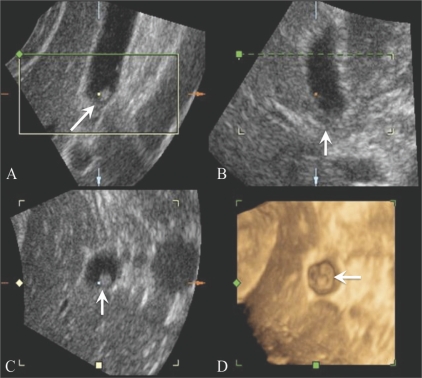
3-D multiplanar viewing of a gallbladder polyp (arrow) in a 59-year-old man. The transverse (A), sagittal (B) and coronal (C) images are perpendicular to each other. The 3D surface-rendered image (D) is also displayed simultaneously

***2. Multiplanar viewing:*** In multiplanar viewing, a 3D voxelbased image must first be reconstructed and then be easily accessible by the display algorithm. Computer user-interface tools allow a selection of planes from the volume, including the oblique plane, to be viewed as reformatted 2D images. Not only do these planes appear similar to those obtained by conventional 2D USG imaging with proper interpolation, but the technique also provides a display of an arbitrary plane that was not possible using conventional 2D USG technique.[4] Three perpendicular planes are displayed on the screen simultaneously, with screen cues as to their relative orientation and intersections, allowing the operator to properly orient the reformatted images [Figure 2]. Several commercially available 3D USG systems already use this technique.

3. ***Volume-based rendering:*** The most widely used volume-based rendering approach is the ray-casting technique,[5] which projects a 2D array of rays on the 3D image. Another common approach is to form a maximum intensity projection (MIP) image by displaying only the voxels with the maximum intensity along each ray.[4] Similarly, a minimum intensity projection (minIP) image can be also reconstructed when only the voxels with the minimum intensity along each ray are displayed.

**Figure 3 (A,B):**
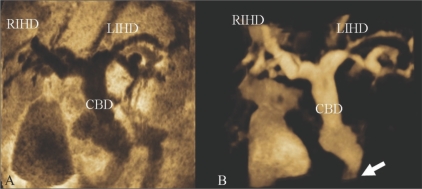
A 70-year-old man with distal common bile duct (CBD) cancer. MinIP (A) and inversion mode (B) images show that the intrahepatic ducts (IHD) and CBD are severely dilated due to a distal CBD lesion (arrow in B). The inversion mode tool transforms anechoic voxels into solid areas. MinIP : minimum intensity projection, RIHD = right intrahepatic duct, LIHD = left intrahepatic duct

Inversion mode is a new post-processing tool that uses a rendering algorithm for the 3D analysis of fluid-filled structures[6] and transforms echolucent structures into solid voxels. Thus, anechoic structures, such as the lumen of the great vessels, bile duct, and gallbladder, appear echogenic on the rendered image, whereas structures that are normally echogenic prior to gray-scale inversion (e.g. bones) become anechoic. Examples are shown in Figure 3.

### Application in the hepatobiliary system

Compared with CT scan or MRI, USG has an advantage in that the scanning plane can be selected more freely than with the other modalities. However, in clinical practice, this is sometimes not possible. For instance, the true coronal plane of the liver is hard to obtain because a part of the liver is sheltered behind the lower ribs. Three-dimensional USG can display information in a manner that has not previously been possible with conventional techniques.[1] Three-dimensional volumetric measurements, including gallbladder measurements to assess gallbladder function, have been made more accurate by recent developments in 3D USG.[7,8] This improved accuracy, especially for organs with an irregular shape such as the gallbladder, also reduces the variability in serial measurements, thus standardizing sonographic procedures. Recently, Kim *et al*, have reported that 3D USG of the gallbladder is clinically feasible and more useful than oral cholecystography or 2D USG alone.[7] Their results showed an excellent correlation between gallbladder ejection fractions measured using 3D USG and the grades of oral cholecystograms. The results indicated the superiority of 3D USG over oral cholecystography for evaluating patients with gallbladder dysfunction and, moreover, demonstrated the clinical usefulness of volumetric analysis by 3D USG for gallbladder evaluation. Volumetric assessment of solid tumor burden is also important in the field of oncology because 3D measurement of tumor volumes gives a more accurate assessment of tumor burden than do traditional unidimensional and bidimensional measurements. This is also true for the monitoring of tumor burden after local ablation treatments such as percutaneous ethanol injection or radiofrequency ablation. Indeed, several reports have proved that volumetric measurement of the tumor with 3D USG is a more accurate tool than traditional 2D measurement.[9] In addition, 3D USG permits three-dimensional visualization of the tumor with its adjacent vessels, imaging of gallbladder pathology and of the structures of the biliary duct, and 4D USG helps guide procedures like biopsies and ablations.[1,10]

## Three-dimensional CT

For the evaluation of suspected hepatic and biliary pathology, CT scan has been the most widely used modality. Recently, multidetector CT scan (MDCT) has been shown to be quite useful, especially for hepatic volume acquisitions, by combining short scan times and narrow collimation with the ability to obtain multiphasic data. These features result in improved lesion detection and characterization. With advances in computer software, 3D applications for hepatic image analyses and displays have been made possible and practical. Kamel *et al*, have discussed the specific types of hepatic and biliary pathology in which MDCT has a significant diagnostic impact.[11]

## Preoperative assessment of complex anatomy

**Figure 4 (A-D):**
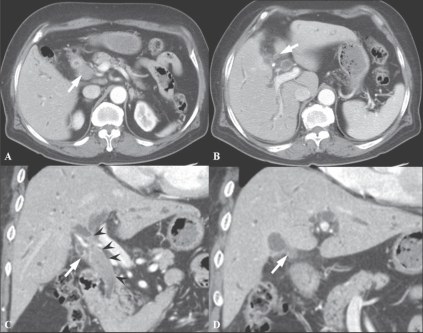
A 72-year-old man with hilar CBD cancer. Axial CT scan (A) during the arterial phase shows a soft tissue tumor in the mid-CBD (arrowheads). At the level of the right portal vein, the axial CT scan image (B) shows tumor infiltration at the orifice of the cystic duct (arrow). Oblique coronal reformation (C) reveals a soft tissue tumor extending from the hilum to the mid-CBD (arrowheads). Coronal MPR image (D) demonstrates involvement of the tumor at orifice of the cystic duct (arrow). These advantages of MPR can create a roadmap for surgeons who are not good at reading axial images

Choi *et al*, performed a study to evaluate the usefulness of multiplanar reconstruction (MPR) images in the assessment of the biliary tree of patients with bile duct cancer. MPRs provide intuitive images and a roadmap for surgeons, displaying the entire length of the bile duct and showing ductal thickening and intraductal masses. According to their results, they could not obtain significantly improved diagnostic performance by adding MPR images to the standard axial images in complex structures like the hepatic hilum. However, they insisted that although MPRs do not increase diagnostic performance in patients with bile duct cancer, they are still valuable in planning therapeutic options and giving confidence to surgical decisions, as well as in allowing a second way of looking at the tumor. MPRs are expected to be very helpful for radiologists with limited experience in hepatobiliary imaging by providing easier-to-read images and views from different angles. Furthermore, one study has shown that when assessing vascular involvement and tumor resectability, higher interobserver agreement was seen in the group that viewed combined axial and MPR images than in the group that viewed only the axial images[12] [Figure 4].

**Figure 5 (A-D):**
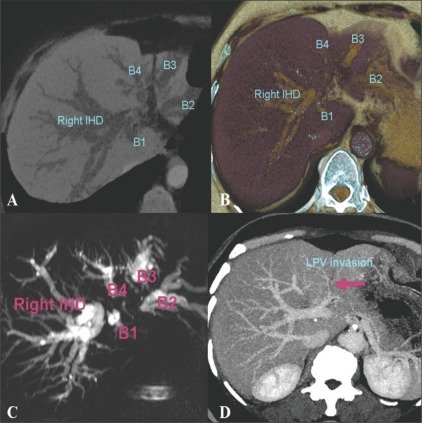
A 69-year-old woman with hilar cholangiocarcinoma. Axial MinIP CT scan (A) and volume rendered (B) images during the portal venous phase, demonstrate dilated and separated intrahepatic ducts. The left intrahepatic duct is involved at the 2nd confluence level which causes separation of the individual left ducts. The MRCP image (C) depicts tumor involvement suggesting a Klatskin IIIb lesion. Axial MIP portal venous phase CT scan (D) shows left portal vein invasion by the tumor, which is depicted as a defect (arrow) MinIP : minimum intensity projection MIP : maximum intensity projection

CT cholangiography or portography using minIP, MIP, and volume rendering images can be useful in the preoperative evaluation of the tumor extent in hilar cholangiocarcinoma [Figure 5].

Recently, Uchida *et al*, described how CT image fusion with 3D reconstructions is used to depict the anatomic structures of the hepatic hilum in detail in the presence of hepatobiliary abnormalities. They illustrated the anatomic features of the hepatic hilum in 3D detail, using a fusion of CT angiographic and CT cholangiographic images; this allows a one-step, comprehensive, noninvasive evaluation of the hepatic hilum. They insisted that high-resolution 3D fusion images will be extremely useful for evaluation of the hepatic hilar anatomy, which is essential for preoperative planning of hepatic and bile duct resection and for liver transplantation.[13]

**Figure 6 (A-D):**
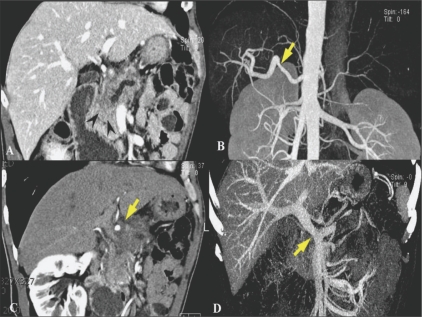
A 68-year-old man with pancreatic cancer. Oblique coronal MPR CT scan image (A) shows an ill-defined infiltrating mass (arrowheads) in the pancreatic head causing upstream pancreatic duct dilatation. Oblique coronal MPR (B) and MIP (C) arterial phase CT scan images demonstrate circumferential soft tissue infiltration around the common hepatic artery with deformity (arrows) suggesting tumor invasion. Coronal portal venous phase MIP image (D) reveals marked narrowing of the main portal vein near the portomesenteric confluence (arrow), indicating tumor invasion into the main portal vein in one plane. MPR : multiplanar reconstruction, MIP : maximum intensity projection

In pancreatic cancer, 3D images allow accurate preoperative local staging, as well as assessment of local resectability, because thin-slice MPR imaging exactly depicts the grade of circumferential involvement and improves the assessment of vascular invasion.[14] With angiographic datasets, displays of the local venous and arterial anatomy can be created, which can provide exact spatial information, show the relation with adjacent structures, and provide other accurate information necessary for surgical planning.[15] Pancreaticography-type images can also be created through advanced rendering [Figure 6].

**Figure 7 (A-D):**
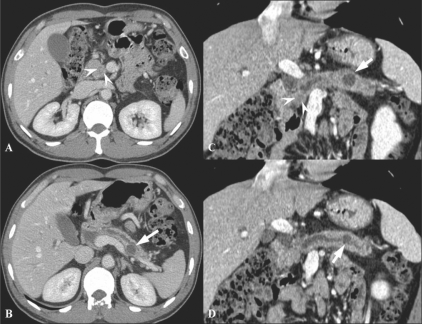
A 44-year-old man with pancreatic head cancer and a pseudocyst in tail. Axial CT scan (A) shows an ill-defined low attenuated mass (arrowheads) in the uncinate process of the pancreas. The main pancreatic duct is diffusely dilated (B) with another cystic lesion in the pancreatic tail (arrow in B). Curved MPR images (C,D) show better appreciation of the tumor (arrowheads in C) and the tail cyst (arrows in C and D), including its communication with the main pancreatic duct (arrow in D)

In the detection of pancreatic carcinoma, MPR images are equivalent to axial images. Curved MPR images improve the depiction of the main pancreatic duct and increase diagnostic performance as compared to axial images alone. Diagnostic performance was significantly improved when both axial and MPR images were used[16] [Figure 7].

Volume-rendered cholangiopancreatography (VRCP) is a 3D image created by using a postprocessing computer algorithm from intravenous contrast-enhanced abdominal CT datasets, without the use of a biliary contrast agent. Johnson *et al.* reported that VRCP in the setting of biliary obstruction due to pancreatic cancer provided valuable 3D information about the intra- and extrahepatic biliary tree, especially with regard to the location and length of the obstruction and the relationship of the intrahepatic ducts to liver metastases; this information was helpful in planning biliary drainage.[17]

## Preoperative local staging of gallbladder cancer

**Figure 8 (A,B):**
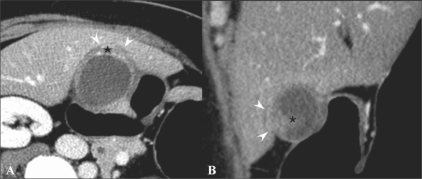
A 54-year-old woman with gallbladder cancer. Axial CT scan (A) shows eccentric wall thickening and a papillary lesion (*) in the fundus of the gallbladder. The fat plane between the liver and the gallbladder seems to be preserved in the axial plane (arrowheads). This lesion was interpretated as T2 on the axial CT image. Oblique coronal MPR image (B) reveals focal hepatic invasion (arrowheads), thus changing the staging and management. Asterisk indicates the papillary lesion

Kim *et al.* reported the diagnostic performance of axial-only images and of combined axial and MPR datasets for differentiating ≤ T2 from ≥ T3 tumors; the combined axial and MPR datasets showed statistically significant greater accuracy (*P* = 0. 0412).[18] They also revealed that combining MPR images with axial images improved the overall accuracy of T-staging of MDCT with axial images (from 71.7% to 84.9%; *P* = 0.0233), especially due to the reduction in partial volume effects, which may be problematic in areas where the gallbladder axis is tangential to the scanning plane [Figure 8]. Furthermore, since the oblique coronal surgical plane can be simulated on MPR, the MPR images may be helpful in surgical planning.[18]

## Assessment of anomalous pancreaticobiliary ductal junction

Using high-resolution multiplanar reconstruction MDCT images it is possible to ascertain where the pancreatic and biliary ducts join, allowing a diagnosis of anomalous pancreaticobiliary ductal junction.[19] The entire course of the common channel itself is not always well seen on axial CT because, in many cases, the channel courses through an area of reduced enhancement, between the pancreatic head and the duodenum;, in some cases, the entire course of the common channel is difficult to see on a single-slice multiplanar reconstruction image; and, often, the common channel is tortuous. In addition, as Sugiyama *et al.* reported, the length of the common channel on CT is not always fully consistent with that found on ERCP.[20] However, MDCT can usually determine the relationship between the confluence of the pancreatic and biliary ducts and the pancreatic parenchyma in most cases. These favorable results may be because of the following two reasons: first, isotropic or nearly isotropic imaging, using axial reconstruction images with a 0.5-mm or 1-mm slice thickness at 0.5-mm intervals over a 260-mm field of view provides sufficiently high resolution in the z-axis for the evaluation of the pancreatic and biliary ducts;[21] second, because of oblique passes of the common bile duct through the pancreatic head and the second part of the duodenum.

## Liver transplantation: Preoperative evaluation of vascular anatomy

**Figure 9 (A-C):**
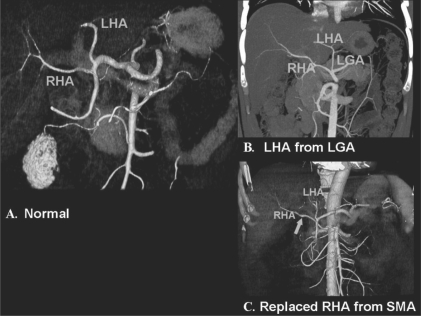
Hepatic arterial anatomy variations. Volume-rendered coronal oblique image (A) shows the classic branching pattern of the HA. The right (RHA) and left (LHA) branches arise from the HA. Coronal oblique MIP image (B) demonstrates the LHA arising from the LGA. Volume-rendered coronal oblique image (C) shows the replaced RHA arising from the SMA, while the LHA arises from the HA. HA: hepatic artery, SMA: superior mesenteric artery, LGA: left gastric artery

**Figure 10 (A-C):**
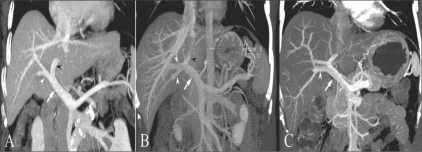
Portal venous anatomy. Coronal oblique MIP images. Image A shows the normal bifurcation pattern of the portal vein; the right and left portal veins (white and black arrowheads) arise from the MPV (arrow). Image B shows a trifurcation pattern; RAAV, RPPV (white arrowheads) and LPV (black arrowhead) arise from the MPV (arrow), simultaneously. Image C shows early branching of the RPPV (arrowhead) from the MPV (arrow), followed by bifurcation into the RAPV and LPV. LPV: left portal vein MPV: main portal vein RAAV: right anterior portal vein RPPV: right posterior portal vein

A comprehensive vascular roadmap facilitates detailed surgical planning and reduces the postoperative complication rate in both, the donor and the recipient.[22] MDCT enables exquisite anatomical detail to be obtained as a result of improvements in spatial and temporal resolution.[23,24] Workstation manipulation of the datas*et al*lows those images to be created that a transplant surgeon can readily understand. [Figures 9 and 10].

## Detection of complications and guidance of procedure

**Figure 11 (A-D):**
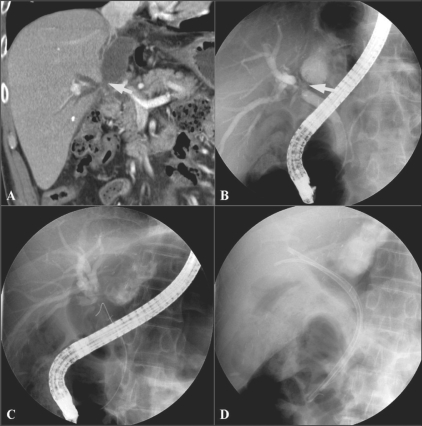
A 52-year-old man who had undergone a liver transplantation presented with fever. Coronal MPR CT scan (A) shows dilated IHDs and a loculated fluid collection near the CHD (arrow). Bile leak at the anastomotic site of the CHD and communication between the biloma and CHD was suspected. ERCP (B) in the same plane, reveals bile leak at the anastomotic site (arrow). Endoscopic CBD stent insertions (C, D) were performed. MPR: multiplanar reconstruction, CHD: common hepatic duct, IHD; intrahepatic duct

**Figure 12 (A-C):**
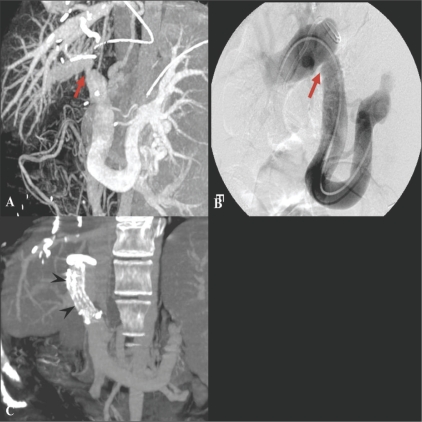
A 45-year-old woman with portal venous stenosis that occurred 8 days after liver transplantation. Coronal oblique MIP image (A) reveals a tight stenosis (arrow) at the portal vein anastomosis. Anterioposterior view of a direct main portal venogram (B) confirms the stenosis (arrow). The stenosis disappeared following metallic stent placement. Follow-up coronal MIP CT scan image (C) shows a patent stent (arrowheads) in the main portal vein

**Figure 13 (A-D):**
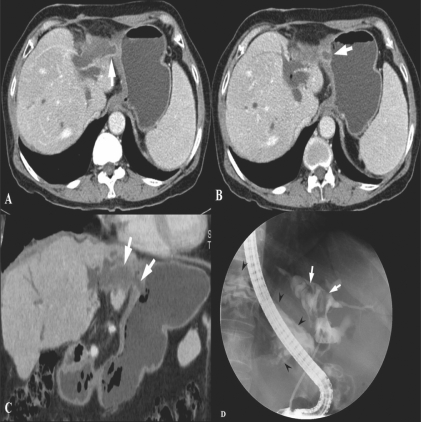
A 68-year-old woman presenting with epigastric pain was found to have a biliary-enteric fistula between the left hepatic duct and gastric cardia. Axial portal venous phase CT scans show balloon like dilated left hepatic ducts and thickening of the adjacent gastric wall. Communication between the dilated duct and the gastric lumen is seen (arrows). Coronal MinIP image (C), demonstrates the fistulous tract (arrows) better. The ERCP image (D) shows severe dilatation of the left duct with multiple cord-like filling defects (arrows) that represent mucin in the duct. The contrast-filled gastric lumen and normal gastric folds (arrowheads) are also observed simultaneously

**Fig. 14 (A-D):**
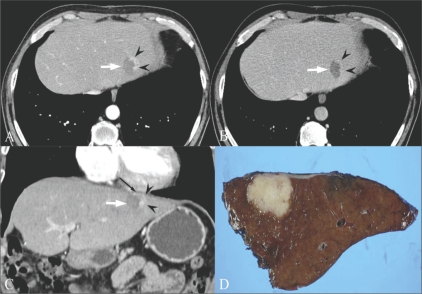
A 55-year-old man was referred due to marginal recurrence of cholangiocarcinoma at the site of a previous radiofrequency ablation. Arterial phase (A) and portal venous phase (B) axial CT scans show a marginally enhancing lesion (arrowheads in A) with wash-out (arrowheads in B) at the periphery of the previously ablated site (arrow) in the lateral segment of the left lobe of the liver. Initially, repeat radiofrequency ablation was considered for this recurrent mass. However, the coronal MPR image (C) revealed close proximity between the suspected viable tumor (arrowheads) and the inferior pericardium (thin black arrow). To avoid thermal damage to the pericardium and to guarantee a safe and clear margin from the viable tumor, the patient underwent surgery. The lesion was separated from the pericardium and pathologically proven to be a recurrent cholangiocarcinoma. This case demonstrates the role of MPR images as a guidance to determine which procedure is appropriate. MPR : multiplanar reconstruction

The incidence of biliary complications has ranged from 11 to 25%. They are a major cause of morbidity following orthotopic liver transplantation and affect graft survival, the duration of hospital stay, recovery, and overall cost of care. The most common complications are biliary leaks, strictures, and stones.[25] Portal vein stenosis or thrombosis occuring during the early posttransplantation period can be devastating, resulting in loss of the graft.[26] Therefore, knowledge of these complications and early detection are important. The development of therapeutic endoscopic and percutaneous radiologic methods has made it possible to manage these complications in a less invasive manner. MPR images can be helpful in determining these therapeutic approaches [Figures 11–14].

## Three-dimensional MRI

### Liver transplantation: Preoperative evaluation of vascular and bile duct anatomy

**Figure 15 (A-C):**
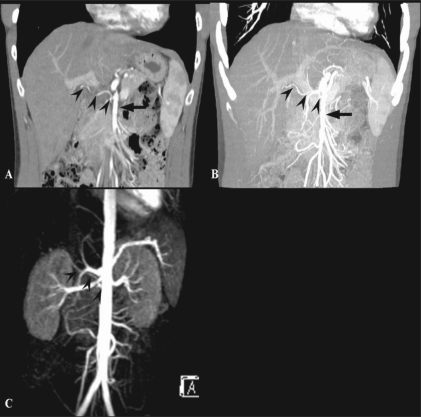
A 35-year-old man with a variation in HA anatomy. Arterial phase MPR (A) and MIP (B) CT scan images show the right HA (arrowheads) arising from the SMA (arrow). MRI (C) also demonstrates a variation in the right HA, Arterial phase MIP MRI also demonstrates the right HA (arrowheads) arising from the SMA (SMA is masked by the aorta). HA: hepatic artery, SMA: superior mesenteric artery

MRI is a useful alternative to CT and has the advantage of being radiation free. Technological breakthroughs in MRI development, such as advances in gradient strength and surface coil sensitivity and the introduction of parallel imaging, have led to the availability of high-resolution MRI with short acquisition times. Several studies have shown that both CTA and MRI angiography (MRA) produce sufficient information of the hepatic vascular anatomy in living liver donor candidates[23] [Figure 15].

**Figure 16 (A,B):**
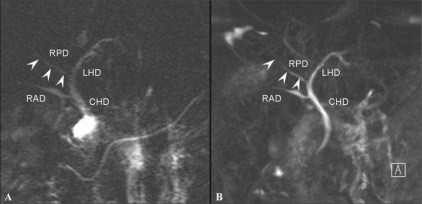
A 35-year-old man with an anatomical variation of the biliary tree. 2D coronal T2W TSE image (TR 2800, TE 1100, FA 150) (A) shows drainage of the RPD (arrowheads) into the left main duct with separate drainage of the RAD into the CHD. 3D coronal T2W (TR 4235, TE 545, FA 180) MIP image (B) demonstrates this with better resolution. RPD: right posterior duct, RPD (arrowheads) is more clearly depicted on 3D MIP image. RAD: right anterior duct, LHD: left hepatic duct, CHD: common hepatic duct

**Figure 17 (A,B):**
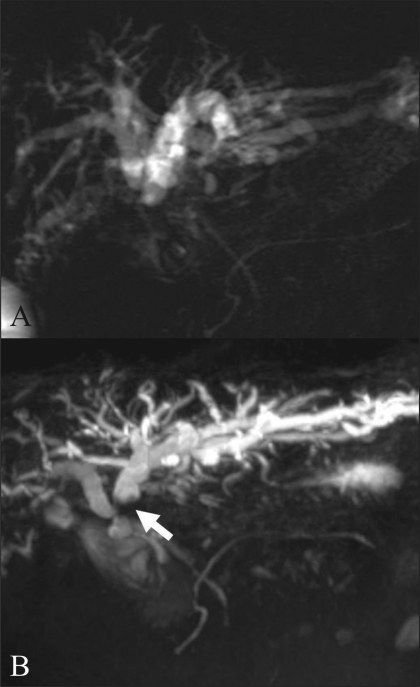
A 67-year-old woman with hilar cholangiocarcinoma, Bismuth type IIIa. 2D thick section RARE MRC (A) demonstrates abrupt narrowing of the proximal CHD and bilateral IHD dilatation. Both secondary confluences are not well-depicted. Navigator-triggered isotropic 3D RARE MRC (B) reveals involvement of the left secondary confluence (arrow). The ovarall image quality of the 3D MRC is better than that of the 2D MRC. RARE: rapid acquisition with relaxation enhancement MRC: magnetic resonance cholangiography CHD: common hepatic duct IHD: intrahepatic duct

Many studies have shown that MRI cholangiography (MRC), using T2W TSE or HASTE sequences, can clearly depict the biliary anatomy, but cannot show all biliary anomalies due to the limited resolution and 2D character of these sequences.[27] However, even though 3D MRC may provide superior image quality as compared to 2D MRC for the evaluation of the extent of disease in malignant biliary obstructions, there has been no statistically significant difference in accuracy[28] [Figures 16 and 17].

### Depiction of the communication between pancreatic intraductal papillary mucinous neoplasm (IPMN) and pancreatic duct

**Figure 18 (A-C):**
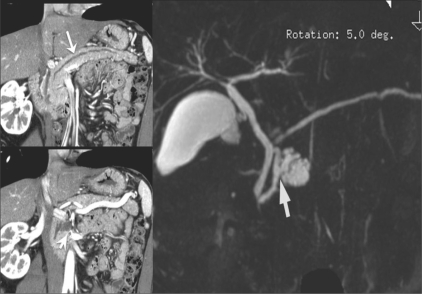
A 60-year-old man with a pathologically proven intraductal papillary mucinous tumor of the pancreas. Two consecutive oblique coronal MPR images (A,B) show a diffusely dilated pancreatic duct and a pleomorphic cystic lesion in the pancreatic head. This suggests communication between the cystic mass and the pancreatic duct. Navigator-triggered isotropic 3D RARE MRCP (C) definitely depicts a pleomorphic, multilobulated cystic mass with ductal communication (arrow), suggestive of a branch duct type intraductal papillary mucinous tumor

In a recent publication, Sahani *et al*,[29] have shown that MDCT combined with 2D curved reformation can provide imaging details similar to MRCP, in patients with IPMN and can show communication of the branch duct–type IPMN with the main pancreatic duct. According to Song *et al*, the diagnostic confidence with MRCP for evaluating the ductal communication of the cystic lesions in 25 patients (25/53, 47%) [Figure 18] with available 2D curved reformation images was higher than with MDCT and MPR images. So, even though MDCT using various postprocessing techniques provides detailed information on cystic structures[30], MRCP is still usually better than thin-section CT scans.[31]

## Summary

There is more to three-dimensional imaging than just pretty pictures. 3D imaging's ability to redefine diagnostic confidence in three-dimensional planes has made it possible to solve many clinical problems. The advantages of 3D imaging are that it allows real-time multiplanar imaging and global depiction of 3D anatomy, there is less operator dependency, and it is more objective. Three-dimensional imaging is especially helpful in hepatobiliary and pancreatic disease evaluation because it is of help in understanding the spatial anatomy and pathology, in enhancing clinical efficiency by providing intuitive images, and in increasing the confidence of accurate targeting in interventions.
